# Editorial: Methodological innovations and translational insights in Early Life Adversity studies

**DOI:** 10.3389/fnbeh.2026.1863849

**Published:** 2026-05-12

**Authors:** Gonzalo Viana Di Prisco, Paul Ruiz

**Affiliations:** 1 Indiana University Indianapolis, Indianapolis, IN, United States; 2 Universidad de la Republica Uruguay, Montevideo, Uruguay

**Keywords:** anhedonia, Early Life Adversity, emotional development, mental health, stress

Early Life Adversity (ELA) events such as neglect, isolation, stress, and physical and emotional abuse impact on brain development and affect behavioral and cognitive processes later in life. ELA has been linked to neuropsychiatric disorders like anxiety and depression and also to addiction, neurodegeneration, and mental health. ELA research can shed light on the biological and psychosocial processes that contribute to increased risk for lifelong mental health challenges. Taken together, the studies in the present topic show that ELA can influence not only how individuals feel, but also how they behave and adapt to challenges later in life.

In this special issue, Suslow et al. shows that childhood emotional neglect is linked to poorer emotion management in adulthood, suggesting that ELA can disrupt the development of emotional intelligence. Likewise, Tran et al. show that adolescents exposed to ELA were more likely to develop anhedonia, a reduced ability to experience pleasure and a key risk factor for depression. Both studies highlight that ELA increases vulnerability to later mental health issues in particular causing emotional difficulties. These emotional difficulties often translate into behavioral consequences. Lin et al. demonstrates that individuals with ELA are more likely to engage in risky sexual behaviors, increasing their likelihood of becoming central transmitters in HIV networks. In a preclinical work Simon et al. shows that social stress during adolescence in rats can lead to binge eating in adulthood, a maladaptive coping strategy linked to emotional distress. All these findings suggest that when ELA disrupts emotional regulation, individuals may turn to unhealthy or risky behaviors as ways of coping. Emotional impairments and behavioral outcomes are indeed closely interconnected. Another important point is the highlight of the role of developmental timing and biological influence as shown by Soerensen et al. They show that prenatal stress by maternal anxiety and depression during pregnancy are associated with increased negative emotionality in children.

Despite the risks, the studies also point to individual differences and potential protective factors. Tran et al. found that higher reward-seeking behavior can reduced the impact of ELA on anhedonia, indicating that some traits can buffer against negative outcomes. Similarly, Suslow et al. hints that positive social satisfaction may have beneficial effects on biological development. These findings suggest that while ELA increases risk, it does not determine outcomes entirely; resilience and environmental support can modify its effects.

The collected studies advance ELA research by moving beyond descriptive associations toward mechanistically informed and methodologically refined models of risk and resilience. Across human and animal designs, the contributions integrate novel biomarkers (e.g., telomere dynamics), behavioral phenotyping, and context-sensitive measures of adversity, while also leveraging longitudinal and multilevel approaches to capture how early exposures become biologically embedded and expressed over time. Importantly, several papers introduce more ecologically valid and translationally relevant paradigms, linking ELA not only to canonical outcomes such as depression and anhedonia, but also to health-risk behaviors, social functioning, and metabolic phenotypes, thereby expanding the phenotypic spectrum of ELA-related vulnerability. A distinctive contribution of this Research Topic is its emphasis on moderators and individual differences—particularly sex, reward sensitivity, and developmental timing—highlighting that ELA effects are conditional rather than uniform. Collectively, these works underscore a shift toward precision models of adversity, where the field is encouraged to move from cumulative exposure indices to process-based, multidimensional, and predictive frameworks.

Overall, the present contributions interconnect to show that emotional neglect can impair emotional intelligence, which may contribute to maladaptive coping behaviors like binge eating or risky sexual activity, while also increasing vulnerability to depression-related symptoms such as anhedonia. At the same time, these processes begin early—even before birth—and are shaped by both biological and environmental factors. ELA sets the stage for later emotional, behavioral, and developmental outcomes, but these effects are complex and influenced by individual differences. By understanding how these factors interact, researchers can better identify intervention points to mitigate the long-term impact of early life stress.

